# Supplementary material for: Novel Driver Strength Index highlights important cancer genes in TCGA PanCanAtlas patients
Source: PeerJ. 2022 Aug 11;10:e13860. doi: 10.7717/peerj.13860 (PMC9375969; doi:10.7717/peerj.13860)
Supplement: Supplemental Information 2 [file peerj-10-13860-s002.zip › COHORTS/genes plots/cohorts/2021_8_16_14_9_distribution_cohorts_ACC.pdf]

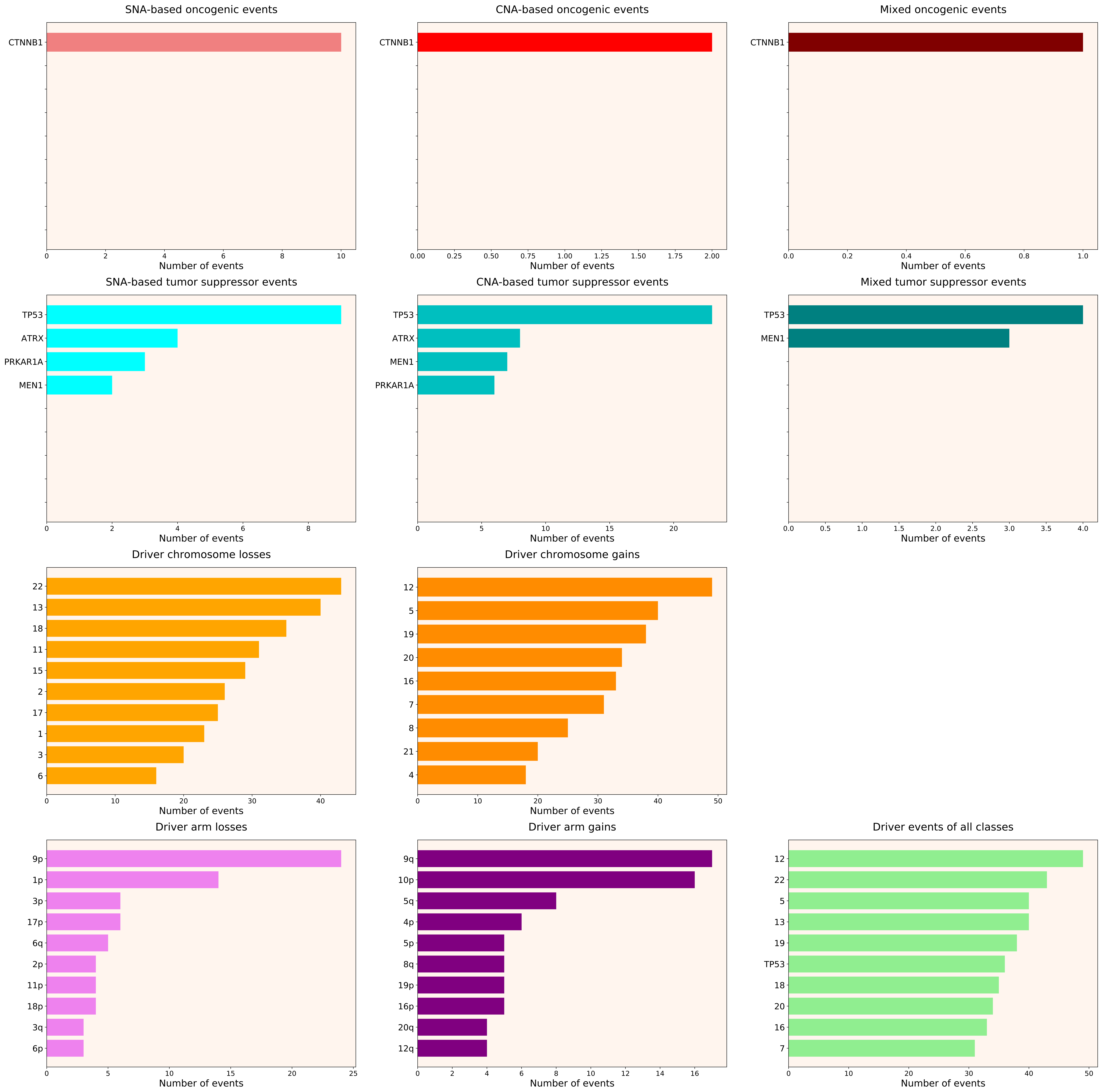
SNA-based oncogenic events
CTNNB1
0 2 4 6 8 10
Number of events
CNA-based oncogenic events
CTNNB1
0.00 0.25 0.50 0.75 1.00 1.25 1.50 1.75 2.00
Number of events
Mixed oncogenic events
CTNNB1
0.0 0.2 0.4 0.6 0.8 1.0
Number of events
SNA-based tumor suppressor events
TP53
ATRX
PRKAR1A
MEN1
0 2 4 6 8
Number of events
CNA-based tumor suppressor events
TP53
ATRX
MEN1
PRKAR1A
0 5 10 15 20
Number of events
Mixed tumor suppressor events
TP53
MEN1
0.0 0.5 1.0 1.5 2.0 2.5 3.0 3.5 4.0
Number of events
Driver chromosome losses
22
13
18
11
15
2
17
1
3
6
0 10 20 30 40
Number of events
Driver chromosome gains
12
5
19
20
16
7
8
21
4
0 10 20 30 40 50
Number of events
Driver arm losses
9p
1p
3p
17p
6q
2p
11p
18p
3q
6p
0 5 10 15 20 25
Number of events
Driver arm gains
9q
10p
5q
4p
5p
8q
19p
16p
20q
12q
0 2 4 6 8 10 12 14 16
Number of events
Driver events of all classes
12
22
5
13
19
TP53
18
20
16
7
0 10 20 30 40 50
Number of events
